# Cervical spondylolisthesis in mucopolysaccharidosis type II

**DOI:** 10.1007/s10072-022-06357-5

**Published:** 2022-08-25

**Authors:** Alessandro Rossi, Giancarlo Parenti

**Affiliations:** 1 Department of Translational Medicine, Section of Pediatrics, University of Naples “Federico II”, Naples, Italy; 2 Telethon Institute of Genetics and Medicine, Pozzuoli, Italy

**Keywords:** Lysosomal storage diseases, Bone, Enzyme replacement therapy, Magnetic resonance imaging, Back pain, Spine

A 14-year-old boy with mucopolysaccharidosis type II (MPS II) presented to the Metabolic Unit with back pain. He was diagnosed with MPS II at age 3 years based on familial history (i.e., elder brother diagnosed with MPS II), clinical features (coarse facies, joint contractures, hepatomegaly, umbilical hernia) and molecular testing (p.Met488Ile and p.Gly489Ala pathogenic variants in the *IDS* gene). He was started with enzyme replacement therapy (ERT) with idursulfase at 3.5 years of age (0.5 mg/kg weekly).

Back pain dated back to about two months before evaluation. The patient displayed full strength in his arms and legs with no sensory deficits or sphincter disturbances. Due to persistent pain in his middle lower back, magnetic resonance imaging (MRI) of the spine was performed, showing grade I spondylolisthesis of C5 and C6 vertebrae (Fig. 1). No signs of spinal cord compression were detected. Treatment with oral nonsteroidal anti-inflammatory drugs was started resulting in symptom resolution in the present case. The patient was included in an MRI follow-up program.

**Fig. 1:**
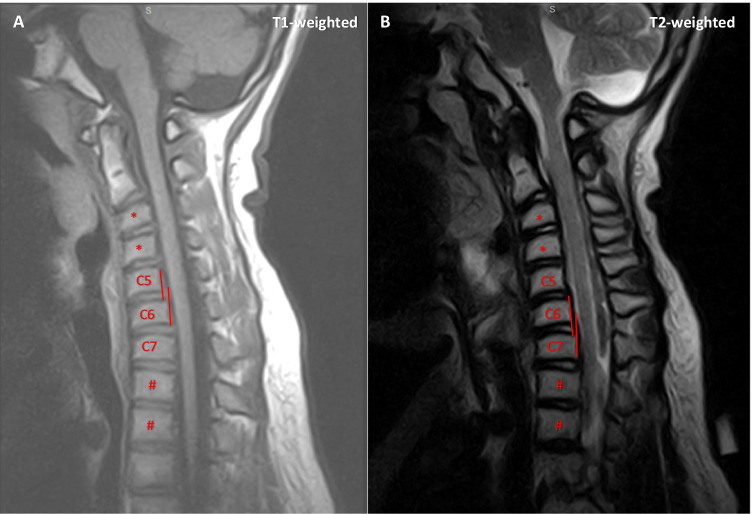
**Magnetic resonance imaging (MRI) of the spine**. (A) T1-weighted sequence. Anterior shift of C5 vertebral body as compared to C6 vertebral body is illustrated. The degree of C5 slip is shown by the two red lines, through the posterior wall of the C5 and C6 vertebral bodies, respectively. The slip is < 25% of the surface of C6 superior endplate (grade I of the Meyerding classification). (B) T2-weighted sequence. Anterior shift of C6 vertebral body as compared to C7 vertebral body is illustrated. The degree of C6 slip is shown by the two red lines, through the posterior wall of the C6 and C7 vertebral bodies, respectively. The slip is < 25% of the surface of C7 superior endplate (grade I of the Meyerding classification). Vertebral “anterior beaking” (asterisk) and “wedge-shaped” vertebrae (hash) are also shown

ERT can correct the extraneuronal features in MPS II. However, bone involvement can still occur including: atlantoaxial instability, spinal cord compression, vertebral “anterior beaking” and “wedge-shaped” vertebrae (Fig. 1)[1]. Spondylolisthesis presents with back pain. It is usually graded using the Meyerding’s classification (grade I-V) [2]. Grade I-II spondylolisthesis typically benefits from conservative treatment. High-grade spondylolisthesis requires surgery. Although thoracolumbar spondylolisthesis has been reported in few cases [3], cervical spondylolisthesis can cause spinal cord compression and should always be investigated and monitored in patients with MPS presenting with back pain.

## Data Availability

Not applicable.
